# The Self-Efficacy of Physicians to Communicate With Patients via Telemedicine in Lieu of Face-to-Face Visits in Light of the COVID-19 Pandemic: An Observational Study

**DOI:** 10.7759/cureus.25739

**Published:** 2022-06-07

**Authors:** Rahul S Rikhy, Janelyn Dela Cruz, Arunima Rattan, Ayesha Bibi, Shahid Rangrej

**Affiliations:** 1 College of Medicine, Saint James School of Medicine, Arnos Vale, VCT; 2 College of Medicine, Washington University of Health and Science, San Pedro Town, BLZ; 3 Research, Saint James School of Medicine, Arnos Vale, VCT

**Keywords:** telemedicine (tm), practice redesign, continuity of care, clinical guidelines, covid-19

## Abstract

Introduction: The coronavirus disease 2019 (COVID-19) pandemic has perpetuated the switch to increased use of telemedicine for initial consultations for physicians out of the necessity of reducing face-to-face contact. It has not been thoroughly studied whether physicians are as self-efficacious in their ability to communicate virtually versus in person considering the natural difficulty of obtaining some objective data points such as those coming from physical examination techniques via telemedicine.

Methods: The Self Efficacy-12 (SE-12) questionnaire, a physician validated objective measure, was used to assess 101 physicians (96% response rate) from 29 specialties for their self-efficacy when communicating with patients when consulting virtually versus in person.

Results: There was a significant 32.43% decrease (p=<0.01) in physician self-efficacy when a patient was evaluated via telemedicine for the first time.

Conclusion: The significant decrease in self-efficacy provides initial evidence that initial consultations should be done in person to maximize physician self-efficacy when communicating with patients.

## Introduction

The role of telemedicine during the coronavirus disease 2019 (COVID-19) pandemic has rapidly adapted to be an effective service for improving access to optimal healthcare [1]. Physicians from different specialties have incorporated digital communication through telemedicine at the expense of regular in-person visits to practice social distancing guidelines [1]. Because of this, the demand for virtual consultations between patients and physicians has increased. Telemedicine has been repeatedly shown in the literature to be an effective form of rendering physician services [2]. However, whether physicians are as self-efficacious with their clinical communication abilities via telemedicine versus face-to-face encounters has not yet been demonstrated. Quantitively measuring physician self-efficacy in communication in a first-contact scenario is significant since telemedicine naturally alters the mechanism of the patient-physician interface. Telemedicine is likely to be used by many medical specialties in a post-pandemic world [3]. Therefore, it is imperative that when developing clinical practice guidelines involving telemedicine we ascertain the most effective method to continue incorporating this vital tool as we move past the current pandemic. Additionally, we will determine the likelihood of a physician further combining patient input regarding their care (shared decision-making) when consulting virtually, which improves patient outcomes [4].

## Materials and methods

Study design

The Saint James School of Medicine Ethics Committee approved this study to commence on 3/23/21. The Self-Efficacy 12 (SE-12) questionnaire [5] was used to determine physician self-efficacy in communicating with patients. SE-12 is a physician-validated objective measure that measures self-efficacy in clinical communication skills. Additional attention was focused on question number 11, "make a plan based on shared decisions between you and the patient," to find out if telemedicine is more or less likely to assist a physician in incorporating shared decision making into their practice. 

A physician practicing within the United States of America must have utilized telemedicine in an initial consultation with a patient and had an in-person visit in the past year to qualify. The physicians' licenses were verified via their national provider identification (NPI) number. During the trial period between May 1, 2021, and August 31, 2021, physicians were recruited via social media groups such as Facebook and personal contacts to participate in the short survey and fill out an original informed consent form available in the appendices section of this article, before participating. one-hundred-and-five physicians were reached, with 101 physicians providing informed consent and moving forward with the study, yielding a 96% response rate. The physicians filled out the SE-12 once for the initial virtual consultations and once for in-person consultations, totaling 24 questions answered per physician. The questions used were from the original study [5], available in the appendices section.

Categorizing physicians into primary care, surgical, or other specialties

When analyzing our data points, we organized the physicians into three categories: 1. Primary care - the three specialties identified in this category are family medicine, internal medicine, and pediatrics, as defined by the American Board of Medical Specialties [6]; 2. Surgical - all specialties eligible for this category are recognized by the American College of Surgeons as surgical specialties, which include cardiothoracic surgery, colon, and rectal surgery, general surgery, gynecology and obstetrics, gynecologic oncology, neurological surgery, ophthalmic surgery, oral and maxillofacial surgery, orthopedic surgery, otorhinolaryngology, pediatric surgery, plastic and maxillofacial surgery, urology, and vascular surgery [7]; 3. Specialties - all other specialties of medicine as recognized by the American Board of Medical Specialties [6].

## Results

**Figure 1:**
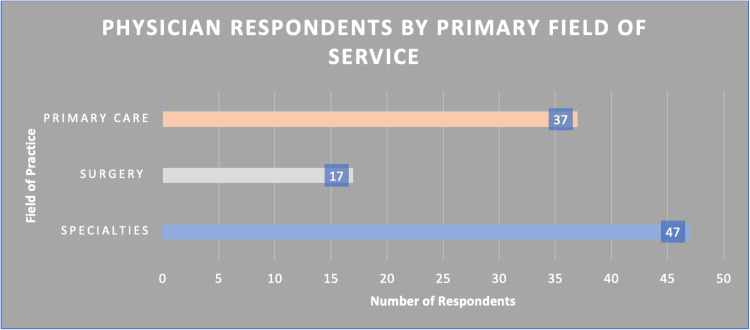
Breakdown of physician respondents by primary field of service

All survey respondents (n=101) were categorized into three primary fields of service: primary care, surgery, and the specialties (Figure 1).

**Figure 2:**
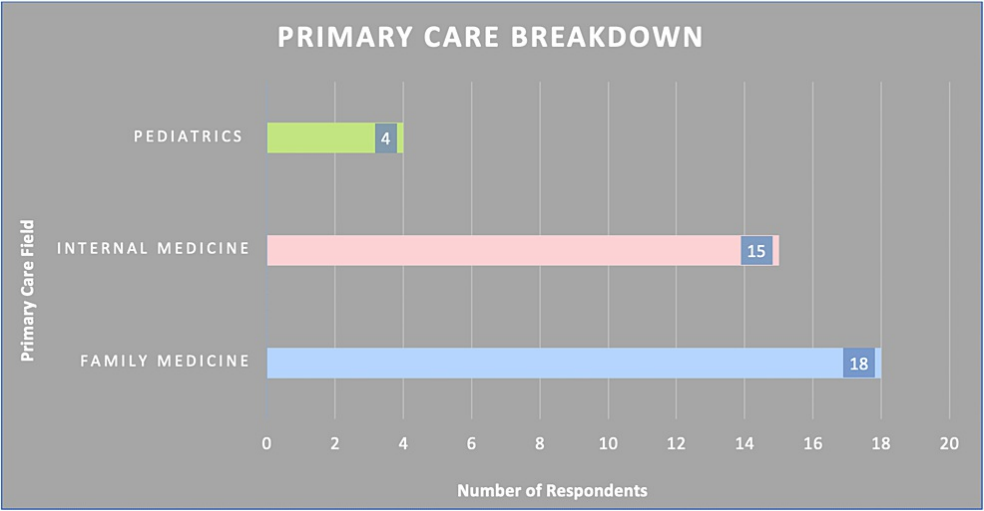
Breakdown of the primary field of service by survey respondent type

Out of 37 primary care respondents, the majority of respondents were Family Medicine physicians (n=18) (Figure 2).

For 37 primary care respondents, the average in-person responses were 9.27 versus 6.43 for virtual consultations. In contrast, a total of 17 respondents were from surgery and had an overall in-person average of 9.59 to a virtual average of 5.39. Maximum respondents in the specialties field were Psychiatrists with a total of eight. Those in the specialties had an in-person average of 9.13 and 6.46 virtually (Table 1). 

**Table 1:** Averages of responses by each primary field of service OB/GYN: Obstetrician-gynecologist

Field of Service	Consultation Method		
	In-Person Average (%)	Virtual Average (%)	Most Respondents & Averages
Primary Care	9.27	6.43	Most Respondents: Family Medicine (n=18) In-person: 9.25 Virtual: 6.74
Surgery	9.59	5.39	Most Respondents: OB/GYN (n=4) In-person: 9.35 Virtual: 5.42 Urology (n=4) In-person: 9.67 Virtual: 6.10
Specialties	9.13	6.46	Most Respondents: Psychiatry (n=8) In person: 9.19 Virtual: 7.65

**Figure 3:**
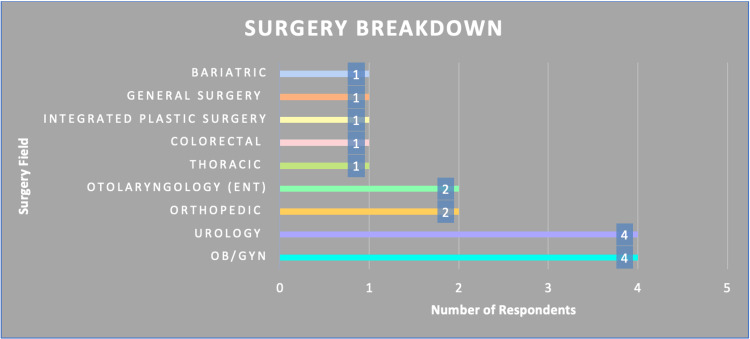
Breakdown of surgery field of service by survey respondent type OB/GYN: Obstetrician-gynecologist

Most respondents were from the fields of obstetrician-gynecologist (OB/GYN) and Urology (n=4) (Figure 3).

**Figure 4:**
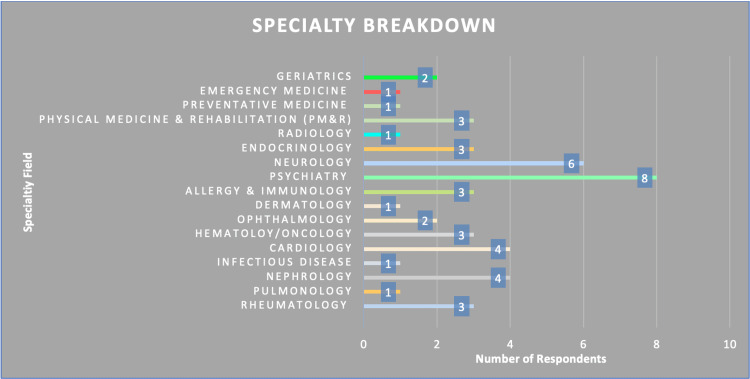
Breakdown of the specialist field of service by survey respondent type

There were 47 respondents in a range of different specialties (Figure 4).

A total of 24 questions were asked in the survey. Physicians were asked 12 questions about in-person consultations and another 12 questions about virtual consultations. Each set of 12 questions of in-person consultations and virtual consultations received a total of 1211 responses from 101 respondents, for a total of 2422. However, two respondents, one in the field of neurology and one in colorectal surgery omitted their responses to question 1 in the virtual consultation questionnaire and question 7 for the in-person consultation questionnaire, respectively. 

The total mean averages of the SE-12 Survey Data for in-person consultations were 9.25 (σ^2^ = 0.96) versus 6.27 (σ^2^ = 3.8) for virtual consultations. A P-value of 1.35x10^-279^ was calculated using a two-tailed T-test (99% CI). A respondent, on average, was not more likely to incorporate shared decision-making in their practice as determined by question 11 on the SE-12 [5], averaging 9.22 for an in-person consultation versus 6.35 for a virtual consultation (Table 2).

**Table 2:** Summative data of all specialties combined

Datapoint	SE-12 (In-person)	SE-12 (Virtual)
Mean	9.25	6.27
Variance	0.96	3.80
Question 11	9.22	6.35
# of responses	n=1211, 96% response rate	n=1211, 96% response rate
P-value, Two-tailed T-test (combined)	1.35x10^-279^	

## Discussion

The most striking difference in the data set is the consistent gap in self-efficacy between all specialties rating their communication ability in consulting a patient in person higher than virtually consulting. The 32.43% global decrease in self-efficacy of communication skills indicates that although telemedicine has been proven within the literature to be an acceptable form of healthcare rendered by physicians, it does not come without its limitations, particularly in the context of an initial consultation. The rationale for this is likely multifactorial. One issue that may perpetuate the disparity in self-efficacy is obtaining objective data such as physical exams. The discrepancy is further perpetuated by the demographic that is most comfortable consulting via telemedicine happens to be psychiatry (7.65) (shown in Table 1), which naturally uses fewer physical exam objective measures than other fields of medicine. Additionally, we did not find that a physician was more likely to incorporate shared decision-making when consulting virtually (6.35) versus in person (9.22) (shown in Table 2). This decrease indicates that the clinical benefit of shared decision-making in patient care was not more likely to be incorporated, regardless of the medium.

Some areas may be improved within telemedicine. Challenges and natural difficulties with telemedicine include the technology required to conduct the visits with quality equipment and connections [8]. The telemedicine program is much more likely to be successful if the technology is designed well from the very beginning in the planning process [9]. As such, proper video conferencing is an essential part of the telemedicine visit requiring adequate equipment on both ends between the physician and the patient [10]. The literature has shown potential security threats while utilizing telemedicine, such as data confidentiality concerns, issues with informed consent, and the potential for hackers to obtain protected health information [11]. Although the Health Insurance Portability and Accountability Act (HIPAA) states that proper authorization is warranted for sharing information between healthcare professionals, different organizations vary within their privacy policies regarding which data points from telemedicine visits are stored [12]. It is imperative to ensure that quality of care is preserved by addressing security concerns and reassessing the standards required for telemedicine by collaborating with experts in information security, which may benefit the security of the technology clinicians utilize [13].

Before COVID-19, physicians did not utilize telemedicine to the extent we see in modern practice. Accordingly, telemedicine became the primary communication medium between doctors and patients during the pandemic. Telemedicine has allowed patients to be assessed while also mitigating the risk for infection control [14]. It has been shown within the literature that patients are generally satisfied with a telemedicine consultation [15]. Patients have expressed several advantages to telemedicine, such as reduced time, immediate feedback, and rapid course of action- including increased access to specialists [16]. Recent advances have been made in evaluating a patient via telemedicine, such as utilizing virtual otoscopes, dermatoscopes, and stethoscopes [17]. However, consultations performed via telemedicine have proven to demonstrate gaps within a physician's capabilities to appropriately react to all situations during an initial consultation, such as an emergency. To illustrate, In acute events such as a stroke, telemedicine cannot consistently substitute the time-critical commencement of therapy for the patient at a remote site; nonetheless, it can aid in symptom identification [18]. 

Telemedicine has been repeatedly shown in the literature to be an effective form of rendering physician services [2]. However, to our knowledge, before this study, the literature has not yet demonstrated whether physicians are as self-efficacious with their communication abilities via telemedicine versus face-to-face encounters. Physician communication is an area of research that researchers must continually study if telemedicine is constantly incorporated instead of face-to-face visits as we move past the pandemic.

Our study does not come without limitations, such as the sample size. Since convenience sampling was used, 101 physicians were the maximum number of physicians recruited within our means within the sampling period of May 1st 2021 to August 31st, 2021. All 101 participants do not necessarily reflect the opinions of all American physicians in the specialties surveyed, as a more extensive study population would be necessary to make such a statement. Additionally, some demographics, particularly the surgical subspecialties, yielded fewer participants. The data presented should be used as a framework for future, sizeable studies to improve upon and increase the power of our findings. One potential bias that should be addressed is selection bias since physicians were approached directly for recruitment into this study. We attempted to minimize this bias by not giving the participants instructions outside of the informed consent form; however, we acknowledge that this may reduce the internal validity of our data, which makes repeat studies essential to corroborate our findings. 

Future research could include replicating our study using new methods that collect comparable data obtained during face-to-face consultations. Such data may consist of remotely assessing muscle tone and strength, evaluating visual fields, and assessing reflexes, which are techniques that may be improved within the telemedicine consultation [18]. Additionally, further research could determine how physician self-efficacy changes over time during follow-up visits, as our study intentionally focused on initial consultations.

## Conclusions

The use of telemedicine can impact patients’ lives, as it offers convenient health care on the patient’s time and schedule. However, physicians must be prudent when deciding the appropriate time to use this tool. Factors that may give rise to this evident decrease in self-efficacy of communication ability during the initial consultation may include the lack of specific objective data points that a physician would typically obtain during a traditional face-to-face consultation. Our study provides guidance for policymakers to consider when creating clinical guidelines for telemedicine and serves as a framework for a larger scale, similar study to improve the precision of our results.

## Appendices

Consent Form

Informed consent form for the participant 

Title: The Self-Efficacy of Physicians to Communicate With Patients via Telemedicine in Lieu of Face-To-Face Visits in Light of the COVID-19 Pandemic: an Observational Study 

The purpose of this study is to identify whether or not a physician is as self-efficacious in their ability to communicate with patients seen via telemedicine in contrast with patients seen via in-person visits, as well as identify the likelihood of the physician incorporating shared decision making with the patient and physician within the plan of care, which will be determined by the 11th question on the SE-12 (Self Efficacy Questionnaire-12).

To qualify, you must be a physician and have had an initial consult with a patient via telemedicine and had an initial consult a patient via a traditional in-person visit. physician status will be verified via the NPPES NPI Registry (https://npiregistry.cms.hhs.gov), and the NPI number will be immediately disposed of.

The objective measure we will use is the SE-12, an accurate measurement validated in studying physicianz self-efficacy in their communication ability with patients. There are 24 questions total, 12 of which ask about consults with patients via telemedicine and 12 asking about interactions with patients via regular in-person consults.

Researchers will always respect the respondent’s privacy. Data will be exclusively accessible only to the listed researchers and immediately deleted after. We will not ask for any information aside from what is outlined in the study. If questions make you feel uncomfortable, you have the right to refuse to answer them. This is a voluntary study, and you may withdraw from it at any time.

Please read and check the following statements below if you agree to participate in the study:

I confirm that I have read and understood the information gathered for the study I have agreed to participate in. I have had the opportunity to consider my rights and ask questions that were answered.

I understand that my participation in the study is voluntary, and I can withdraw my participation at any time.

I agree to take part in the study.

Electronic signature of participant:_______________________________________________

Questionnaire 

"How certain are you that you are able to successfully …

1. Identify the issues the patient wishes to address during the conversation?

2. Make an agenda/plan for the conversation with the patient?

3. Urge the patient to expand on his or her problems/worries?

4. Listen attentively without interrupting or changing focus?

5. Encourage the patient to express thoughts and feelings?

6. Structure the conversation with the patient?

7. Demonstrate appropriate non-verbal behavior (eye contact, facial expression, placement, posture, and voicing)?

8. Show empathy (acknowledge the patient’s views and feelings)?

9. Clarify what the patient knows in order to communicate the right amount of information?

10. Check the patient’s understanding of the information given?

11. Make a plan based on shared decisions between you and the patient?

12. Close the conversation by assuring, that the patient’s questions have been answered?"

Reference no. [5]
